# Altered temporal, but intact spatial, features of transient network dynamics in psychosis

**DOI:** 10.1038/s41380-020-00983-1

**Published:** 2021-01-18

**Authors:** Danhong Wang, Xiaolong Peng, Andrea Pelletier-Baldelli, Natasza Orlov, Amy Farabaugh, Shahin Nasr, Hamdi Eryilmaz, Maurizio Fava, Avram J. Holmes, Joshua L. Roffman, Hesheng Liu, Daphne J. Holt

**Affiliations:** 1 Athinoula A. Martinos Center for Biomedical Imaging, Department of Radiology, Massachusetts General Hospital, Harvard Medical School, Charlestown, MA, USA; 2 Department of Radiology, Tongji Hospital, Tongji Medical College, Huazhong University of Science and Technology, Wuhan, China; 3 University of North Carolina at Chapel Hill, Chapel Hill, NC, USA; 4 Department of Psychiatry, Massachusetts General Hospital, Harvard Medical School, Boston, MA, USA; 5 Department of Psychology, Yale University, New Haven, CT, USA; 6 Department of Neuroscience, Medical University of South Carolina, Charleston, SC, USA; 7 Beijing Institute for Brain Disorders, Capital Medical University, Beijing, China

## Abstract

Contemporary models of psychosis suggest that a continuum of severity of psychotic symptoms exists, with subthreshold psychotic experiences (PEs) potentially reflecting some genetic and environmental risk factors shared with clinical psychosis. Thus, identifying abnormalities in brain activity that manifest across this continuum can shed new light on the pathophysiology of psychosis. Here, we investigated the moment-to-moment engagement of brain networks (“states”) in individuals with schizophrenia (SCZ) and PEs and identified features of these states that are associated with psychosis-spectrum symptoms. Transient brain states were defined by clustering “single snapshots” of blood oxygen level-dependent images, based on spatial similarity of the images. We found that individuals with SCZ (*n* = 35) demonstrated reduced recruitment of three brain states compared to demographically matched healthy controls (*n* = 35). Of these three illness-related states, one specific state, involving primarily the visual and salience networks, also occurred at a lower rate in individuals with persistent PEs (*n* = 22), compared to demographically matched healthy youth (*n* = 22). Moreover, the occurrence rate of this marker brain state was negatively correlated with the severity of PEs (*r* = −0.26, *p* = 0.003, *n* = 130). In contrast, the spatial map of this state appeared to be unaffected in the SCZ or PE groups. Thus, reduced engagement of a brain state involving the visual and salience networks was demonstrated across the psychosis continuum, suggesting that early disruptions of perceptual and affective function may underlie some of the core symptoms of the illness.

## Introduction

Studies conducted over the past decade have revealed that schizophrenia (SCZ) is associated with disruption of the coordinated functioning of distributed brain networks [1–4]. These disruptions have been commonly identified by measuring fluctuations in low-frequency blood oxygen level–dependent (BOLD) signal in resting-state functional magnetic imaging (rs-fMRI) data [5–8]. Rs-fMRI data are typically examined using a “static” analysis, in which “functional connectivity” is estimated by measuring correlations of magnitudes of BOLD signals in distinct brain regions. Such static connectivity investigations have detected a wide range of alterations in functional connectivity in individuals with SCZ, within fronto-parietal, default mode, ventral attention, and somatosensory networks [9–11]. Although replicable differences in static connectivity between healthy and SCZ groups have been found [12], recent work has suggested that correlations of BOLD signals over minutes do not reflect the rapid changes in brain connectivity that may be most affected in psychotic illness [13–15]. Specifically, recent methodological advances have revealed that human brain activity is characterized by continuous, rapid shifts in activity across a large number of complex, varied networks, rather than a prolonged engagement of a small number of networks [16–19].

To leverage this new understanding of brain connectivity, investigations of the rapid shifts in BOLD signal correlations occurring across the brain have been recently conducted in SCZ. These studies have reported that individuals with SCZ show lower (or higher [20]) rates of switching among functional “states,” reductions in the duration of time spent in cohesive states of regional co-activation, and decreased variation in states, compared to healthy subjects [21–25]. However, recent evidence suggests that findings of these “dynamic” studies that examine correlations of BOLD signals in short time windows are affected by unaddressed confounds related to sampling variability [26].

An alternative approach to measuring transient brain activation is to examine absolute co-activation levels of BOLD signal across the brain at each time point [27]. This method allows for a more direct investigation of the magnitude of activity of functional networks and their interactions, without relying on temporal correlations of BOLD signal in short time windows [26]. Here we used this approach to measure the configurations of functional networks, and the temporal and spatial properties of activity patterns of the brain, in individuals with SCZ and demographically matched healthy controls (HC). Critically, we used the same method in a second cohort of subjects: young adults with persistent subthreshold psychotic symptoms or what are commonly referred to as “psychotic experiences” (PEs) [28] and a group of demographically matched healthy youth (HY) without such symptoms. This second sample allowed us to determine whether any of the patterns observed in SCZ were also found in individuals with PEs, while avoiding the confounds that typically limit the interpretation of neuroimaging findings in SCZ, such as treatment with antipsychotic medication [29, 30].

Although, on average, only a small percentage of individuals with PEs will eventually develop clinical psychosis, the persistence of PEs over time substantially increases such risk (5–25 fold) [28, 31, 32]. Furthermore, recent evidence supports the model that PEs are on a biological continuum with clinical psychosis [33–37]. For example, recent studies have identified changes in brain structure and function in individuals with PEs that are similar to those observed in SCZ [38–42]. Similarities across findings in SCZ and PE groups suggest that such common findings may reflect manifestations of psychosis-related pathophysiology (and are less likely to result from effects of treatment or chronic illness in SCZ). Thus, in this study, we aimed to identify the changes in dynamic brain states that manifest across the psychosis spectrum, in both SCZ and PE groups.

## Materials and methods

### Participants

Three datasets were analyzed in this study. All participants provided written informed consent in accordance with the guidelines of the Institutional Review Boards of Partners Healthcare (Datasets I and III) and Harvard University (Dataset II). All images were collected on 3T Tim-Trio scanners (Siemens, Erlangen, Germany) with 12-channel phased-array head coils.

Dataset I included 35 patients with DSM-IV–diagnosed SCZ (age 41.80 ± 9.01; 8 female) and 35 age, sex, and head motion–matched HC (age 39.49 ± 10.43; 8 female, see Table 1) [43]. One to two resting-state (eyes open) scans (372 s/run) were obtained from each subject. Functional images were acquired using a gradient-echo echo-planar pulse sequence (TR = 3000 ms, TE = 30 ms, flip angle = 85°, 3 × 3 × 3 mm voxels, FOV = 216). Structural data included a multi-echo T1-weighted magnetization-prepared gradient-echo image (TR = 2200 ms, TI = 1100 ms, TE = 1.54 ms, flip angle = 7°, 1.2 × 1.2 × 1.2 mm). As expected, the SCZ group had a significantly lower mean IQ and years of education than the HC group.

Dataset II included 130 enrolled college students (age 19.5 ± 1.3; range 18–24; 91 female) with elevated scores on either a measure of depression (Beck Depression Inventory total score >5, or item #9 > 0) or PEs (Peters et al. Delusions Inventory (PDI) total score >7). Among these subjects, 22 consistently showed moderately elevated PDI scores (total PDI score >4 over two time points 1 year apart) and were included in the PE group (age 19.23 ± 1.07; 8 female). Twenty-two subjects who showed consistently low PDI scores (<4, at two time points 1 year apart) were included in the HY group (19.27 ± 1.28; 8 female). The imaging protocol was identical to that of Dataset I. The PE and HY groups were matched in gender, age, years of education, and head motion (see Table 1).

Dataset III included 20 young healthy subjects (age 29.6 ± 5.3; range 24–40; 10 female). The imaging protocol was identical to that of Datasets I and II. For this cohort only, each subject was scanned twice, once with eyes open and once with eyes closed. These data were used to examine the effects of visual input on the outcome measures of this study.

See the Supplementary methods for additional details.

### Preprocessing

Rs-fMRI were preprocessed using a previously described analysis pipeline [44], which included: (1) projecting the data from the subject’s native space to FSL MNI152 space and then onto the FreeSurfer fsaverage6 surface space; (2) linear detrending and band-pass filtering (0.01–0.08 Hz); (3) regressing nuisance variables including the global signal, head motion parameters, and their first temporal derivatives [45]; (4) spatial smoothing with a 6 mm FWHM Gaussian curve; (5) down-sampling the BOLD fMRI data to a mesh of 642 vertices in each hemisphere; and (6) temporal normalization to the signal extracted from each vertex.

### Clustering-based single frame dynamic analysis

**Fig. 1:**
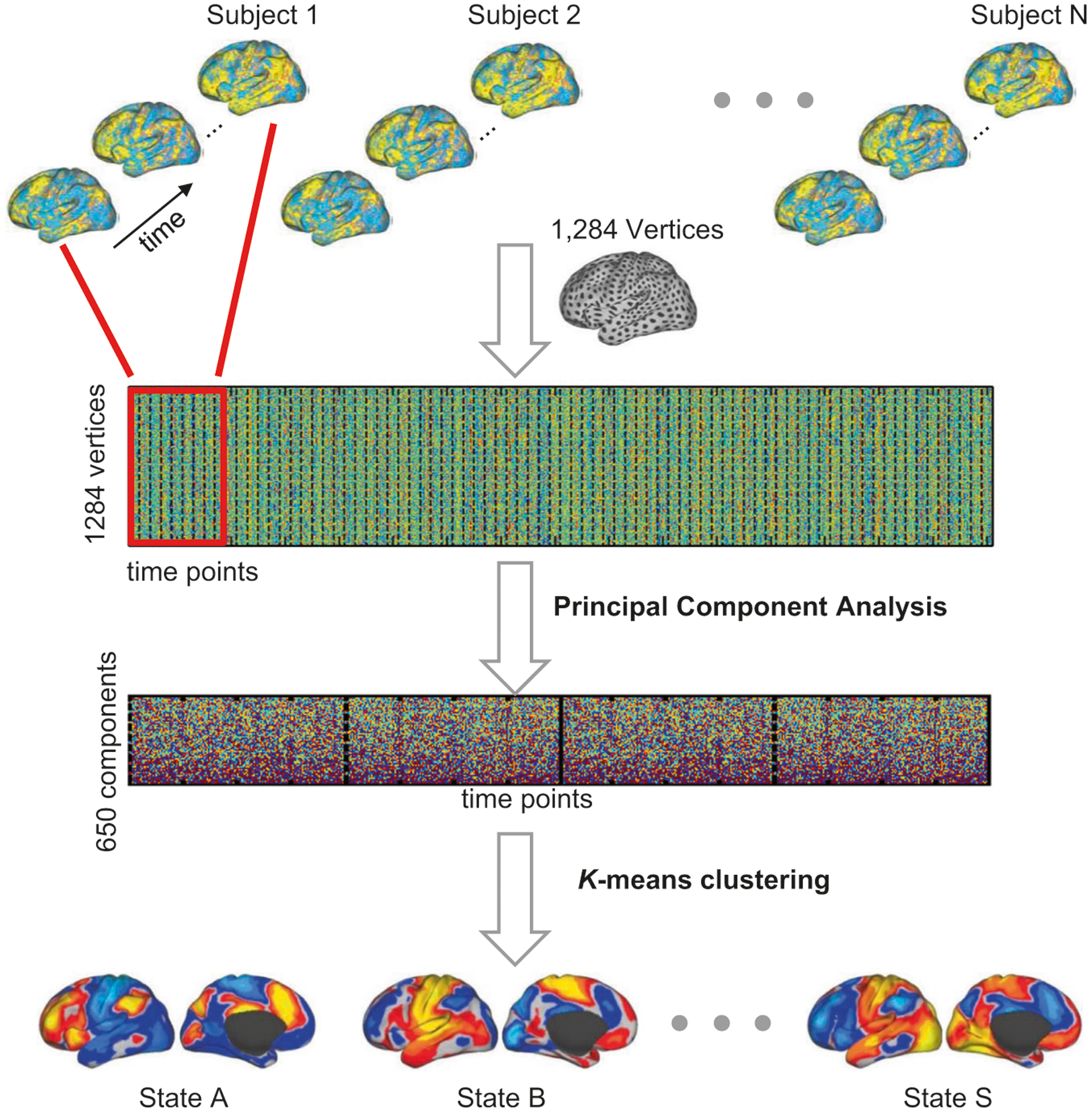
Strategy for estimating brain network states from resting state fMRI. BOLD images were clustered into brain states according to their spatial patterns, i.e., images with similar spatial patterns were clustered together. Specifically, the preprocessed fMRI data of each individual subject was projected to the FreeSurfer fsaverage3 surface space, which consisted of 1284 vertices in total. The data of different time points of each subject were aligned to construct a spatiotemporal matrix (indicated by the red rectangle). The data of all subjects were then concatenated and temporally standardized. Principal component analysis (PCA) was applied to the concatenated data matrix to reduce the dimensionality of data to 650, which could explain more than 99% of the variance of the original data. The *k*-means clustering algorithm was then performed to classify the fMRI frames into a certain number of clusters (e.g., 19 clusters in this study). The fMRI frames assigned to the same cluster were averaged to generate the brain state map.

We applied a *k*-means clustering algorithm to classify BOLD images into different groups based on their spatial similarity (using a cosine similarity metric) (Fig. 1), which has been previously employed to temporally decompose resting-state networks into multiple spontaneous co-activation patterns [27]. The cluster centroids were interpreted as unique “brain states.” Each BOLD image was defined by its signal values across 1284 surface vertices. The preprocessed BOLD signals of all subjects were concatenated and temporally standardized. To improve computational efficiency, we used a principal component analysis to reduce the dimensionality of the feature space to 650, which could explain 99% of the total variance in the rs-fMRI. The optimal cluster number was selected according to the test–retest reliability of the resulted brain states in 100 young healthy subjects scanned using the same scanning protocol as for our data (see Supplementary Fig. S1). The solutions with 11, 14, 19, 31, 35 clusters were relatively reliable. In this study, we focused on 19-states estimation for further analyses.

## Results

### Rate of occurrence of specific brain states was reduced in individuals with SCZ

Dynamic brain states were defined by clustering the single volumes/images of fMRI data based on their spatial similarity (Fig. 1), i.e., images with similar spatial patterns were clustered together, representing a brain state. Here, the term “states” only refers to brain activity patterns rather than cognitive states. Nineteen “brain states” were derived from clustering the resting-state images from SCZ and HC groups (Supplementary Fig. S2). The probability of occurrence was not uniformly distributed across these states, i.e., some states appeared more often than others. The occurrence rate of each state was calculated as the percentage of frames assigned to the state.

**Fig. 2:**
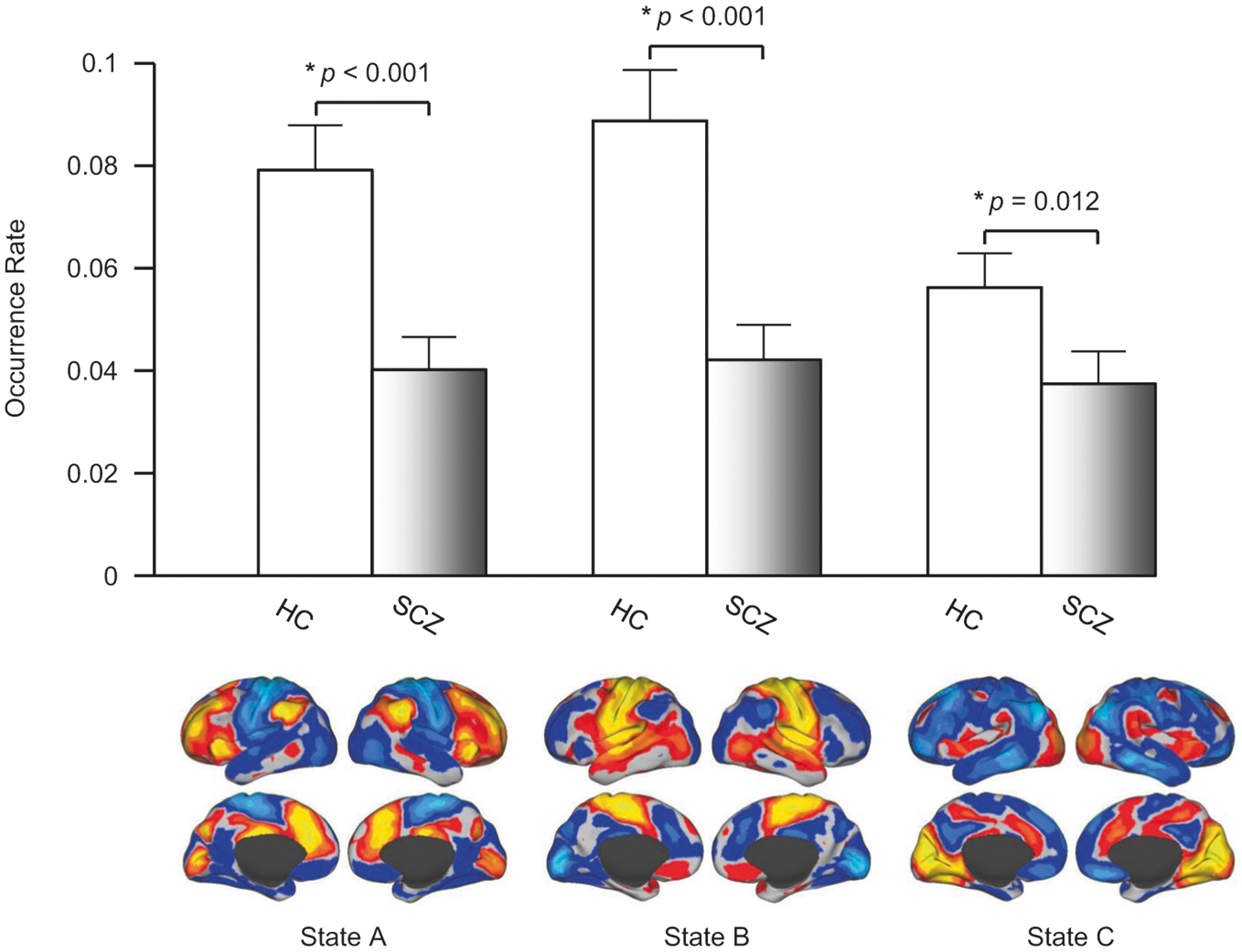
The occurrence rate of three brain states was significantly reduced in patients with SCZ. Nineteen brain states were identified in the resting-state data from 35 patients with SCZ and 35 healthy controls. Three states demonstrated a significantly reduced rate of occurrence in the patients with SCZ compared to the controls (*p* < 0.001, *p* < 0.001, *p* = 0.012, two-tailed *t*-test, respectively). The maps of these brain states in the healthy controls are displayed in the lower panel. One state demonstrates strong activation in the fronto-parietal control network but deactivation in sensorimotor areas. The second state demonstrates the opposite pattern. The third state involves strong activation in the visual cortex and the insula but deactivation in the default network. See also Supplementary Fig. S2 for the maps of the 19 clusters identified in this dataset and Supplementary Fig. S9 for the results using different cluster numbers (cluster number = 11, 14, 17, 19, 21).

Among the 19 states, we identified three states (States A, B, and C; see Supplementary Fig. S2) that demonstrated a reduced occurrence rate in SCZ, compared to HC (*p* < 0.001, *p* < 0.001, *p* = 0.012, respectively, FDR-corrected for multiple comparisons, Fig. 2, see also Supplementary Fig. S3 for individual data points). No states showed an increased occurrence rate in SCZ compared to HC. Maps of these states were compared to seven canonical functional networks previously described [46] (see Supplementary Fig. S4). State A was characterized by strong activation in the fronto-parietal network (FPN) and deactivation in the motor-sensory network (MN). State B had the opposite pattern, i.e., deactivation in the FPN and strong activation in the MN. State C involved strong activation in the visual network (VN) and the salience network (SN), including the insula and angular gyrus, and deactivation in the default mode network. In the SCZ group, the occurrence rate of these three states was uncorrelated with PANSS positive (*p* > 0.66) or negative (*p* > 0.32) symptom scores, or dose of antipsychotic medication (*p* > 0.17).

### The spatial distribution of brain states did not differ between SCZ and HC

Although three states occurred less often in SCZ than in HC, the spatial maps of these states appeared to be similar between the two groups (Spearman correlations between the state maps of two groups: *r* = 0.94, 0.90, 0.92, respectively, see Supplementary Fig. S5). To further examine this similarity, we derived brain states from an independent dataset, the 100 healthy subjects that were used to determine the optimal cluster number (i.e., the test–retest dataset). Maps of States A, B, and C derived from the test–retest dataset resembled those derived from the HC and SCZ groups (Spearman correlations with the maps from HC: *r* = 0.94, 0.90, 0.84 for States A, B, and C, respectively; correlations with maps from SCZ: *r* = 0.91, 0.81, 0.77, respectively). We then used the states derived from the test–retest dataset as cluster centers. In the HC and SCZ groups, we calculated spatial distance (Euclidean distance) from each frame to these cluster centers. For all three states, there was no difference in Euclidean distance from the frames to the cluster centers between the SCZ and HC groups (all *p* > 0.05). These observations indicate that for each state, images from both groups are equally distributed around the cluster center defined in an independent dataset, confirming the spatial similarity of these images.

### Occurrence rate of State C was significantly reduced in PE

**Fig. 3:**
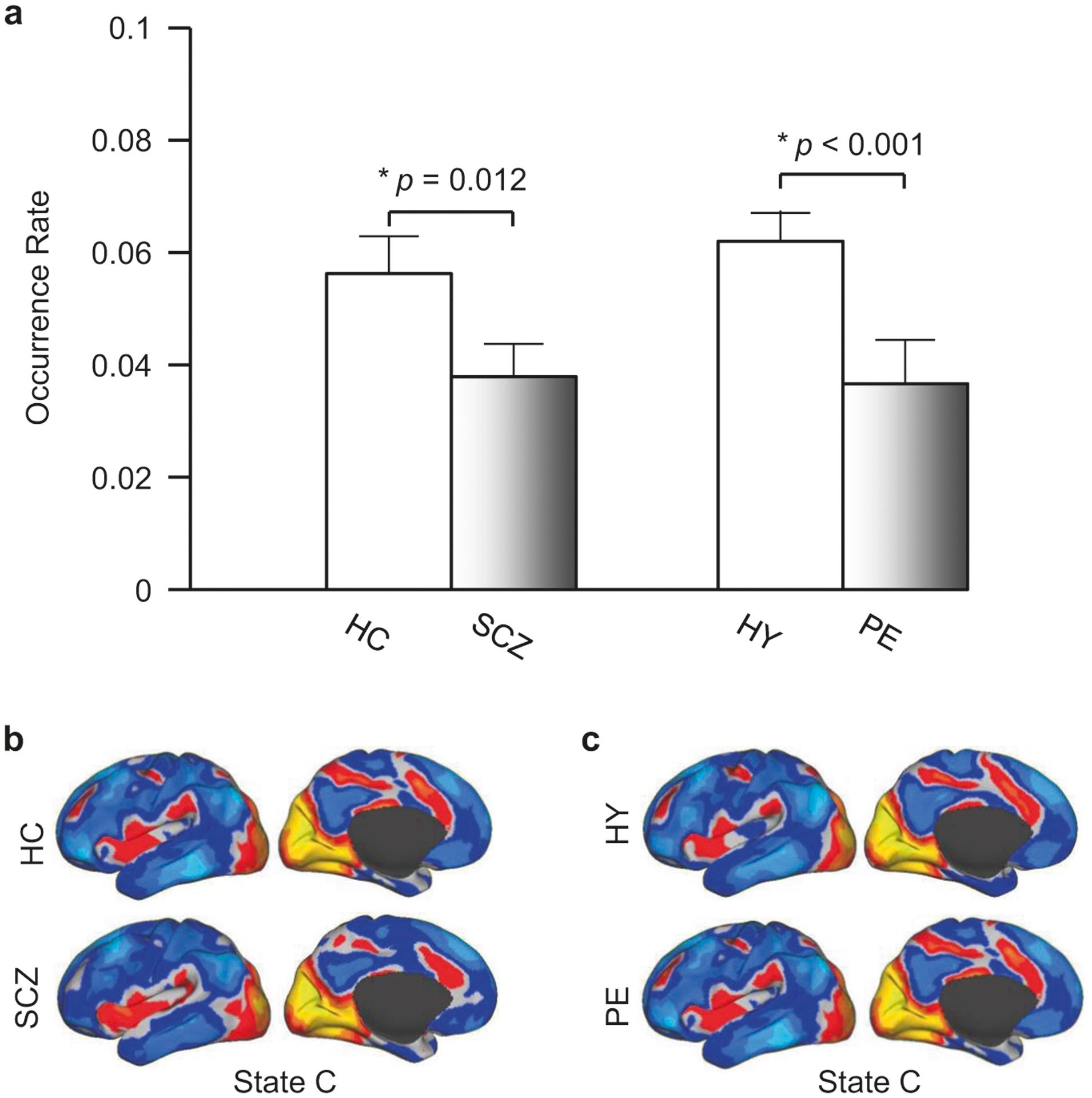
A psychosis-linked “brain state” is mainly comprised of the visual cortical and salience networks. **a** Brain State C, which included activation of the visual cortex, insula, and dorsal anterior cingulate cortex, and deactivation in the default mode network, demonstrated a significantly reduced rate of occurrence in the SCZ and PE groups compared to controls (*p* = 0.012, *p* < 0.001, respectively, two-tailed *t*-test). **b** and **c** The spatial map of State C showed no differences between the SCZ and HC groups, or between the PE and the HY groups (Spearman spatial correlation: *r* > 0.90 for both), indicating that spatial characteristics of this brain state are preserved (but its temporal characteristics are significantly altered) in the SCZ and PE groups.

To identify neural correlates of psychosis that are not confounded by medication effects or history of illness, we also derived 19 states from the rs-fMRI data of the PE and the HY groups. The 19 states were identical to those described above. We found that only one state, State C, differed between the PE and HY groups. This state occurred at a significantly lower rate in PE than in HY (*p* < 0.001, Fig. 3). However, the occurrence rates of State A and State B did not differ between the PE and HY groups (*p* = 0.16, *p* = 0.20, respectively).

The spatial map of State C also did not differ between the PE and HY groups (Spearman correlation: *r* = 0.910, comparison of distance from frames to cluster center between the two groups: *p* = 0.79). These observations indicated that, similar to our findings in SCZ, the spatial characteristics of these dynamic brain states are preserved in individuals with PEs.

### The occurrence rate of brain State C was associated with the severity of PEs

**Fig. 4:**
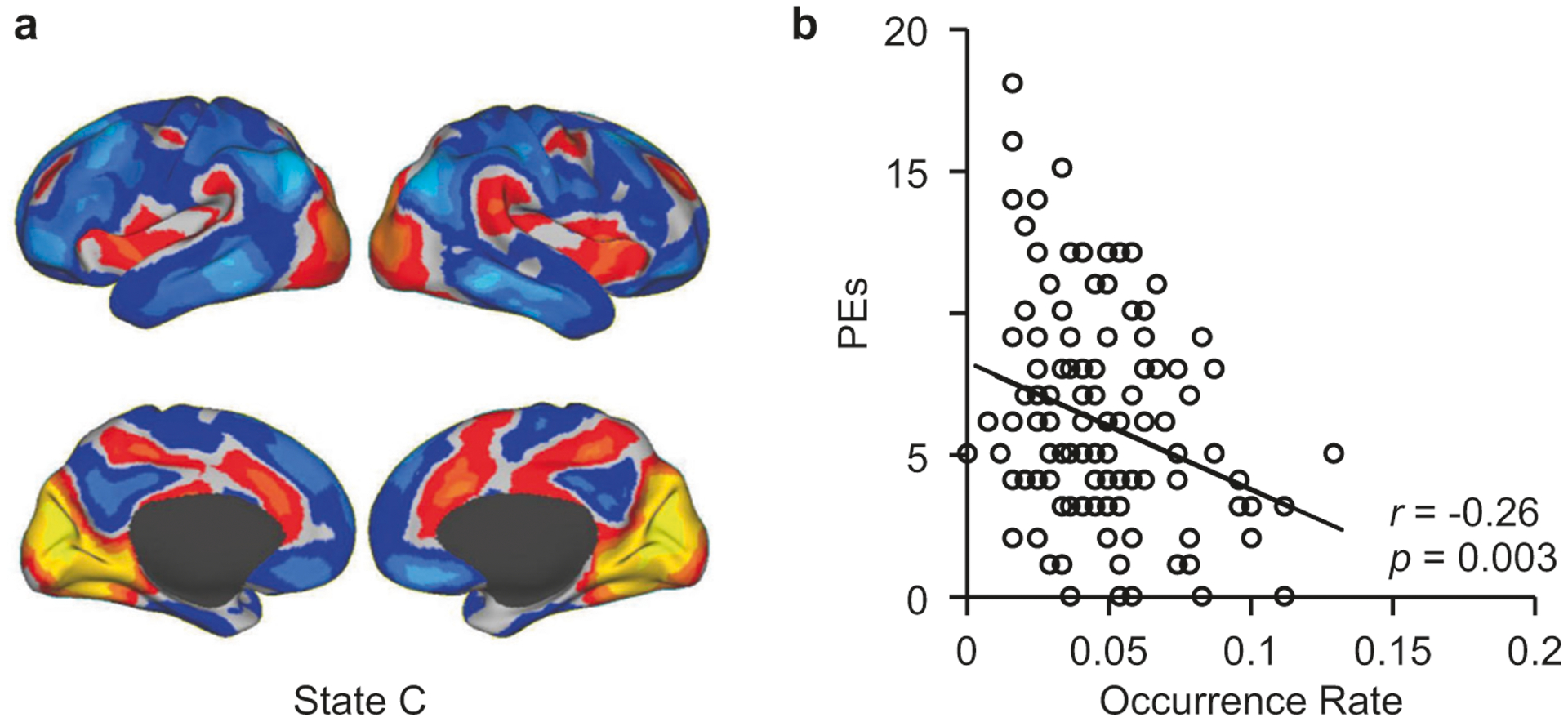
Occurrence rate of the psychosis-related state is associated with the severity of subthreshold psychotic symptoms. Within the full young adult cohort (*n* = 130), the occurrence rate of State C showed a significant correlation with PDI score (*r* = −0.26, *p* = 0.003, explaining 6.7% of the variance in PEs), indicating that the loss of State C is associated with the severity of subclinical psychotic symptoms. A similar correlation between the loss of State C and psychotic symptom severity was not observed in the SCZ group, possibly due to the fact that all of the patients with SCZ enrolled in this study were being treated with antipsychotic medications at the time of data collection.

State C occurred at a lower rate in both SCZ and PE, compared to their controls, suggesting a specific association of this state to psychosis across a spectrum of severity. Thus, we examined whether the occurrence rate of State C could predict symptom severity in the full cohort of young adults that included the PE and HY groups, plus an additional 86 subjects with a low or intermediate level of PEs. Within this cohort (*n* = 130), the occurrence rate of State C showed a small correlation (*r* = −0.26, *p* = 0.003) with the number of PEs, as reflected by the subjects’ total PDI score, indicating that the loss of State C is associated with the level of severity of PEs (explaining 6.7% of the variance in PEs; Fig. 4).

### Static functional connectivity analyses

To investigate how the loss of dynamic brain states is related to changes in conventional “static” functional connectivity, we selected three brain regions that were most activated in States A, B, and C and then computed functional connectivity between these selected seed regions and the rest of the brain (Supplementary Fig. S6). The static connectivity maps of these seed regions resembled the maps of dynamic brain states (all *ps* < 0.001; see Supplementary Table S1). Moreover, compared to HC, the SCZ group showed reduced connectivity (*p* < 0.05, FDR corrected, Supplementary Fig. S6) between the seeds and their connected regions, indicating that reduced occurrence of these states in patients with SCZ may be associated with reduced “static” functional connectivity between brain regions that are co-activated in these states. Similarly, compared to HY, the PE group also showed a trend of reduced connectivity (but did not reach significance after FDR correction) between a seed in the visual cortex and its connected regions (Supplementary Fig. S6). This comparison of analysis approaches in the same cohort suggests that many of the prior reports of diminished functional connectivity in SCZ may be partially attributable to a reduction in the rate of recruitment of certain networks. Indeed, we further found that occurrence rates of States A, B, and C were correlated with static connectivity among the regions co-activated in these states (Supplementary Fig. S7).

### Potential confounding factors

Analyses of data collected in healthy subjects scanned twice during rest, with eyes closed or open (Dataset III), indicated that our findings were not related to reductions in visual input in the SCZ or PE groups (see Supplementary results and Supplementary Fig. S8). In addition, we found that our results were not related to the selection of the number of clusters (see Supplementary results and Supplementary Fig. S9).

## Discussion

In this study, measurement of the occurrence rate of “brain states,” i.e., co-activated brain areas during one acquisition of rs-fMRI (collected over 3 s), revealed three brain states that occurred at a lower rate in SCZ compared to HC. Of these three states, one also occurred at a diminished rate in individuals with persistent PEs. Moreover, the occurrence rate of this specific brain state was negatively correlated with the severity of PEs in a larger group of youth. In contrast, the spatial distribution of this state was unaffected in both the SCZ and PE groups.

### Reduced engagement of FPN, MN, and VN–SN in established psychotic illness

Our results indicate that SCZ is associated with diminished recruitment of states involving the FPN, MN, and VN–SN. These results are consistent with and extend findings of prior neuroimaging studies of SCZ. Altered activation of the FPN in SCZ is a common finding of prior studies [10, 47] and has been linked to impairments in cognitive control, decision making, and the ability to initiate and persist in goal-directed behavior [48–52]. Similarly, altered sensorimotor and visual processes have been frequently reported in SCZ [53–55]. Prior studies have also revealed abnormalities in the SN in psychotic illness [56–59]. The current results suggest that a selective failure to appropriately recruit certain “brain states” that involve these networks may be present in SCZ.

### Reduced engagement of the VN–SN is also evident in individuals with persistent PEs

A diminished occurrence rate of the VN–SN state was also found in the PE group. Moreover, in the larger cohort of 130 youth (of which the PE and HY groups were subsets), reduced occurrence rate of this state was related to the severity of PEs. These results are consistent with prior evidence for a role of the SN and the insula in particular, a key node of the SN, in the pathophysiology of psychotic symptoms [60] (i.e., the replicated associations found between the activity or volume of the insula and psychotic symptoms in SCZ [61–67]). Although the mechanisms underlying these associations remain unclear, the current data taken together with evidence that the insula coordinates responses of widely distributed sensory and attentional networks and mediates transitions (“switching”) between externally directed and introspective processing [56, 68–70] suggest that impairments in SN-mediated transitions may occur in psychosis [56, 57]. Also, the insula is involved in selecting or prioritizing sensory information for the purpose of generating appropriate responses to that information [70–72]. Thus, our findings suggest that reduced recruitment of VN and SN may be associated with an impairment in this selection process, potentially contributing to the development of positive symptoms [57, 73].

### Model of progressive alterations in sensory, attentional, and higher-order neural systems in psychosis

Our finding of selectively impaired engagement of the SN–VN in the PE group, with the additional loss of function of the MN and FPN in SCZ, is suggestive of a progressive involvement of these networks in the pathophysiology underlying psychotic illness. We speculate that an initial disruption of sensory-salience processing at an early, subthreshold stage of illness may be followed by changes in the MN and FPN at later stages in the evolution of the illness. This model is consistent with studies showing that very early “psychosis risk” states are often characterized by the presence of mild sensory processing or cognitive changes that may involve inappropriately ascribing “salience” to incoming sensory information due to spontaneous neural signals that are independent of environmental stimuli (e.g., “basic symptoms” [74], “anomalous experiences” [75], or “aberrant salience” [76, 77]). For most, PEs are transient, often stress related and do not recur [78]. However, for a high-risk subset, these symptoms are distressing and persistent and, in some, associated with clinical progression. Progression may occur due to ongoing, persistent disruptions of sensory and salience processing, which ultimately interferes with cognitive and basic motor processes and function of the associated networks. Alternatively, the additional changes (the reduction in occurrence of States A and B) found in SCZ here, relative to those with PEs, could represent a long-term consequence of having the illness (e.g., the effects of chronic treatment with antipsychotic medication, a lack of employment or social activity). Further longitudinal work is required to test these competing models, by tracking activity of these brain states during sequential stages of illness. If such a model of progression is confirmed, low engagement of the SN–VN could represent a marker of a very early, at-risk state.

### Psychotic experiences: associated risk for clinical psychosis and relationship to other prodromal states

PEs have been studied primarily in non–help-seeking, general population samples with prevalence rates conservatively estimated at ~7%. The overall risk associated with PEs for transition to clinical psychosis is estimated at ~8% [35, 36], but some studies report higher transition rates (e.g., ~25% [37]). The risk of transition depends on a number of factors such as the degree of persistence of the PEs, the presence of affective symptoms, and environmental factors [28, 79].

In contrast, the “clinical high risk” (CHR) category of the psychosis prodrome, which is also primarily defined by the presence of attenuated psychotic symptoms, includes only those who have become help-seeking and distressed, and show recent clinical deterioration [80]. Thus, the CHR state is associated with a much higher level of risk for developing psychotic illness than PEs [81]. Also, both PEs and the CHR state, while increasing risk for psychotic illness, are associated with an even greater likelihood of having or developing a non-psychotic affective or anxiety disorder [28, 82]. Given the high levels of heterogeneity within these two overlapping categories and their association with transdiagnostic risk for neuropsychiatric illness overall, objective measures that can determine who are most vulnerable of those with these risk factors are clearly needed.

### Limitations

A limitation of this study is that, in order to maximize feasibility and recruitment success, the members of the PE group were not clinically assessed for the presence of psychotic disorders. However, it is unlikely that any of the subjects of this study were acutely psychotic given that none were taking antipsychotic medications and all were fully enrolled in college at the time of the study. In addition, the cross-sectional nature of the study’s design limits inferences regarding the relationship between the findings in the PE and SCZ groups; follow-up longitudinal studies can further test the model of illness progression suggested by these results.

It is not yet known whether the changes observed here in the occurrence of these transient brain states in the SCZ and PE groups reflect a relatively reversible impairment in neural responsiveness or synchrony, or a more enduring change in the integrity of the neurons within these networks or of their connections. However, the relationships between the occurrence of brain states and static connectivity suggest that changes in these brain states may be associated with enduring changes in functional coupling and network function. This hypothesis can be directly tested in future studies.

## Supplementary Material

Supplementary

## Figures and Tables

**Table 1:** Characteristics of the participant groups.

A. Demographically matched healthy controls (CON) and individuals with schizophrenia (SCZ) (Dataset I)			
	CON (*n* = 35)	SCZ (*n* = 35)	*P* value
Age (years)	39.49 ± 10.43	41.80 ±9.01	0.32
Gender	77.14% male	77.14% male	>0.90
PANSS Total		72.00 ± 12.72	
PANSS Positive		17.29 ±5.58	
Symptoms Subscale			
PANSS Negative		20.35 ±4.84	
Symptoms Subscale			
PANSS General		34.35 ± 6.55	
Symptoms Subscale			
Head motion (FD)	0.076 ±0.05	0.089 ± 0.06	0.30
IQ	111.76 ±9.49	101.35 ±11.40	<0.01
Years of education	17.21 ±2.18	12.86 ± 2.44	<0.01
B. Demographically matched HY and PE subjects (Dataset II)			
	HY (*n* = 22)	PE (*n* = 22)	*P* value
Age (years)	19.27 ±1.28	19.23 ±1.07	0.90
Gender	63.64% male	63.64% male	>0.90
PDI total score	2.09 ± 1.48	8.36 ±2.52	<0.001
Head motion (FD)	0.0453 ± 0.023	0.0423 ±0.016	0.612
Years of education	13.23 ±1.31	13.23 ±1.15	>0.90

*CON* healthy control group, *SCZ* schizophrenia group, *PANSS* Positive and Negative Syndrome Scale, *FD* framewise displacement (in mm), *HY* Healthy youth group, *PE* psychotic experiences group, *PDI* Peters et al. Delusions Inventory.
