# Supplementary material for: Altered temporal, but intact spatial, features of transient network dynamics in psychosis
Source: Mol Psychiatry. 2021 Jan 18;26(6):2493–503. doi: 10.1038/s41380-020-00983-1 (PMC8286268; doi:10.1038/s41380-020-00983-1)
Supplement: Supplementary file 1 — Supplemental material [file 41380_2020_983_MOESM1_ESM.docx]

**Supplemental Materials** for Wang et al, “Altered temporal, but intact spatial, features of transient network dynamics in psychosis”

**I. Supplementary Methods:**

**Data collection**

Three datasets were examined in this study. All participants provided written informed consent in accordance with guidelines set by the Institutional Review Boards of Partners Healthcare (Datasets I and III) and Harvard University (Dataset II). Subjects were excluded if they had any contraindications for having an MRI scan (e.g., metal implants, claustrophobia, pregnancy) or a history of head trauma. All images were collected on 3T Tim Trio scanners (Siemens, Erlangen, Germany) with 12-channel phased-array head coils.

*Dataset I.* This dataset included 35 patients with DSM-IV diagnosed SCZ (age 41.80 ± 9.01; 8 female) and 35 age- and sex-matched HC (age 39.49 ± 10.43; 8 female) ^1^. The patients were recruited and characterized clinically by the MGH Schizophrenia Clinical and Research Program; diagnoses were determined using the Structured Clinical Interview (SCID) for DSM-IV ^2^. The healthy controls (who were without a history of psychiatric, neurologic or serious medical disorders, or substance abuse during the previous 6 months) were recruited via advertisement. One to two resting-state (eyes open) scans (6 m 12 s per run) were obtained from each subject. All data were collected on a 3T Tim Trio scanner (Siemens, Erlangen, Germany) using a 12-channel phased-array head coil at the Athinoula A. Martinos Center for Biomedical Imaging in Charlestown, MA. Whole brain coverage including the entire cerebellum was achieved with slices aligned to the anterior commissure-posterior commissure plane, using an automated alignment procedure, ensuring consistency among subjects. Subjects were instructed to stay awake, keep their eyes open, and minimize head movement; no other task instruction was provided.

Functional images were acquired using a gradient-echo echo-planar pulse sequence (TR = 3,000 ms, TE = 30 ms, flip angle = 85°, 3 × 3 × 3 mm voxels, FOV = 216 and 47 slices collected with interleaved acquisition with no gap between slices). Structural data included a high-resolution multi-echo T1-weighted magnetization-prepared gradient-echo image (TR=2,200 ms, TI=1,100 ms, TE=1.54 ms for image 1 to 7 .01ms for image 4, flip angle = 7°, 1.2 × 1.2 × 1.2 mm and FOV = 230). Positive and negative symptoms of schizophrenia were assessed by a trained rater in each SCZ subject using the Positive and Negative Syndrome Scale (PANSS) ^3^ at the time of scanning. There were no significant differences between the SCZ and HC groups in gender, age and head motion (see **Table 1**). However, as expected, the SCZ group had a significantly lower mean IQ and years of education than the HC group.

*Dataset II.* This dataset included 130 young adults (age 19.5± 1.3; range 18-24; 91 female; all enrolled college students) with elevated scores on either a measure of depression (Beck Depression Inventory (BDI) total score > 5, or item #9 > 0) or psychotic experiences (Peters et al Delusions Inventory (PDI) total score > 7); these cut-offs were chosen to identify young adults with mild, subthreshold symptoms of depression or psychosis (~ the top 20-50% of the distribution of scores among college students) for longitudinal follow-up (at 6-month intervals using on-line assessments) in an ongoing study of psychopathology in college students ^4^. These subjects were recruited to this study via a parent study that aimed to characterize the level and types of psychopathology in college students using self-report symptom assessments delivered during in-person mental health screenings conducted at several Boston-area colleges ^4^. Among the 130 subjects, 22 consistently showed moderately elevated PDI scores (total PDI score > 4 over two time points one year apart) and were included in the PE group (age 19.23 ± 1.07; 8 female). 22 subjects who showed consistently low PDI scores (< 4, at two time points one year apart) were included in the HY group (19.27 ± 1.28; 8 female). All data were collected on a 3T Tim Trio scanner (Siemens, Erlangen, Germany) using a 12-channel phased-array head coil located at the Center for Brain Science (CBS) at Harvard University in Cambridge MA. The imaging protocol was identical to that of Dataset I; one to two resting-state (eyes open) scans (6 m 12 s per run) were obtained from each subject. There were no significant differences between the PE and HY groups in gender, age, years of education and head motion (see **Table 1**).

*Dataset III.* This dataset included 20 young healthy subjects (age 29.6±5.3; range 24-40; 10 female). All data (n = 20, 10 female) were collected on a 3T Tim Trio scanner (Siemens, Erlangen, Germany) using a 12-channel phased-array head coil at the Athinoula A. Martinos Center for Biomedical Imaging in Charlestown, MA (the same scanner as Dataset 1). Imaging parameters were identical to Datasets I and II. One to two resting-state scans (6 m 12 s per run) were obtained from each subject at each scan session. For this cohort only, each subject was scanned twice, once with eyes open and once with eyes closed. These data were used to examine the effects of visual input on the outcome measures of this study.

**Data processing**

Structural data were processed using FreeSurfer version 5.3.0. Surface mesh representations of the cortex from each individual subject’s structural images were reconstructed and registered to a common spherical coordinate system. The structural and functional images were aligned using boundary-based registration within the FsFast software package (http://surfer.nmr.mgh.harvard.edu/fswiki/FsFast). The preprocessed resting-state BOLD fMRI data were then aligned to the common spherical coordinate system via sampling from the middle of the cortical ribbon in a single interpolation step. FMRI data of each individual were registered to the FreeSurfer cortical surface template (fsaverage6) that consists of 40,962 vertices in each hemisphere. A 6-mm full-width half-maximum (FWHM) smoothing kernel was then applied to the fMRI data in the surface space.

**Identifying optimal cluster numbers**

The optimal cluster number was selected according to the test-retest reliability of the resulted brain states. Specifically, we examined the test-retest reliability of brain states in 100 young healthy subjects obtained from the Brain Genomics Superstruct Project^5^, who were scanned using the same scanning protocol as for our SCZ and HC data. Half of the resting runs were randomly selected from each subject and assigned to the test group; the other half were assigned to the re-test group. The k-means clustering (20 iterations) was performed in the test group and the re-test group respectively, using cluster numbers ranged from 4 to 40. Reliability was then estimated as the mean spatial similarity between the corresponding states derived from the test and re-test group. To obtain a stable estimate, this data splitting procedure was repeated 100 times. Mean reliability across the 100 repetitions and standard error were plotted (see Supplementary **Fig. S1**). Several local maxima on the curve were identified, suggesting that the solutions with 11, 14, 19, 31, 35 clusters were relatively reliable.

**Seed-based static functional connectivity analysis**

For the static seed-based functional connectivity analyses, resting state fMRI data were preprocessed using the procedures previously described^6^, which included (1) slice timing correction (SPM2; Wellcome Department of Cognitive Neurology, London, UK), (2) rigid body correction for head motion with the FSL package, (3) normalization for global mean signal intensity across runs, and (4) bandpass temporal filtering (0.01– 0.08 Hz), head-motion regression, whole-brain signal regression, and ventricular and white-matter signal regression.

We selected the most activated vertex in each brain state and then defined a seed region using the 10 vertices closest to that vertex. Resting-state functional connectivity was then computed between the seed region and the rest of the brain. Specifically, time courses of the seed vertices were averaged, then Pearson’s correlations were calculated between the seed time course and the time courses of each vertex on the brain surface. Spatial similarity between brain state maps and static FC maps were then evaluated using Spearman correlations.

**Spatial correlation between brain maps**

Spearman correlations between two maps were calculated using MATLAB (<https://www.mathworks.com/help/stats/corr.html>). To assess the potential impact of spatial dependence between neighboring vertices on these correlations, we have applied the Durbin Watson test ^7^ using MATLAB (<https://mathworks.com/help/stats/dwtest.html> and https://mathworks.com/help/stats/durbin-watson-test.html). Specifically, we performed a repeated (n=1000) random sampling of 10% of the vertices and computed the correlation coefficient in these subsets of the vertices. For each subset, the Durbin-Watson test was performed to estimate the spatial dependence. Correlation coefficients were z-transformed and averaged across the 1000 iterations. In all spatial correlations reported here, the values of the Durbin-Watson statistic were close to 2, with p-values > 0.05, suggesting that there was no significant spatial autocorrelation in the subsets.

**II. Supplementary Results:**

**Reduced occurrence rate of state C was not due to changes in visual input**

State C, which was reduced in rate of occurrence in both the SCZ and PE groups, includes the visual and salience networks. To address the possibility that non-specific effects, such as lower levels of arousal ^8^ or blinking rates in the SCZ and PE groups, may have influenced these results, we studied 20 healthy subjects that were scanned during rest twice, with their eyes open and with their closed, respectively, and estimated the 19 states. State C showed a similar occurrence rate during the two conditions (p =0.145), suggesting that it is insensitive to levels of visual input. Among the 19 states, only one state (State H) showed a significant difference in occurrence rate between the eyes open and closed conditions (p=0.006, **Fig. S8**), but this state showed no alterations in the SCZ or PE groups. Thus it is unlikely that the individuals with SCZ and PE closed their eyes or were less attentive than controls and that our findings were related to this potential confound.

Given that both State H and State C involve the visual cortex, we also examined whether State C and State H tend to typically transition from one to another in time. We found that State H and State C did not show a high probability of transitioning from one to another. The transition probability from State C to State H ranked #5 among all 19 possible transitions from State C, and the transition probability from State H to State C ranked #7 among all 19 possible transitions.

**Alternative clustering did not impact the primary findings**

Importantly, the loss of the three states in the SCZ group could be reliably observed when we clustered the data into different number of states (11, 14, 17, 19 or 21 states, **Fig. S9**), suggesting that our findings were not driven by a particular selection of cluster number.

**III. Supplementary Tables:**

**Table S1. Spatial similarity between state maps and static FC maps**

|  | FC map of HC | FC map of SCZ |
| --- | --- | --- |
| State A | R=0.587 | R=0.301 |
| State B | R=0.923 | R=0.759 |
| State C | R=0.870 | R=0.862 |

All p-values < 0.0001

**IV. Supplementary Figures:**


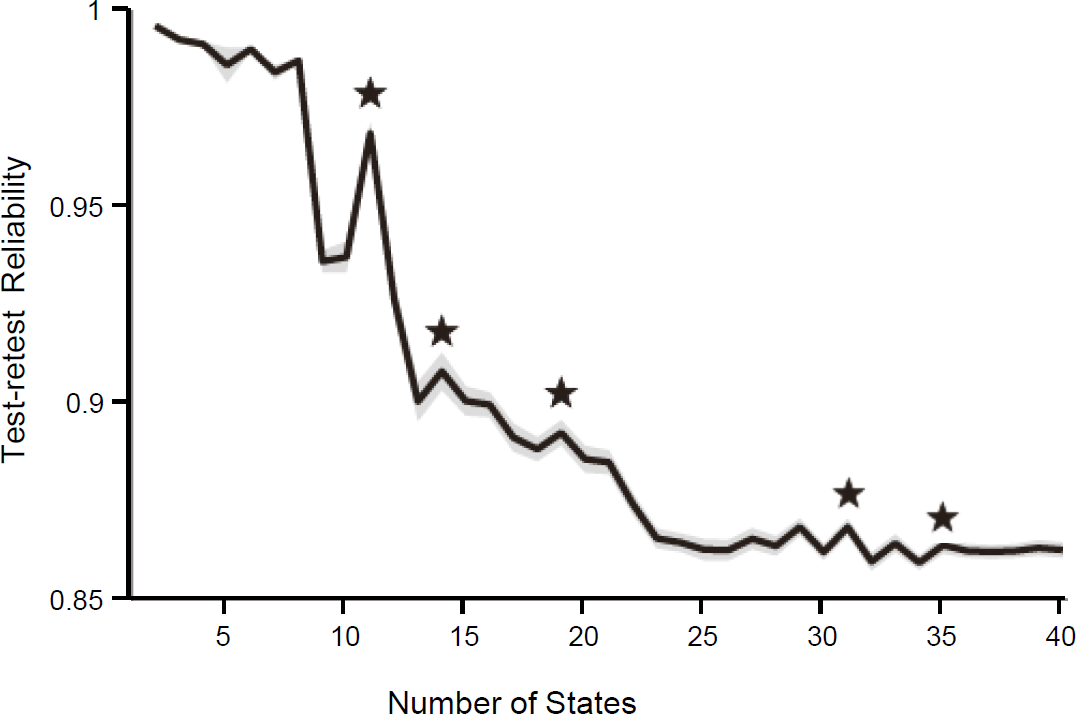


**Figure S1.** **Determining the optimal cluster number based on test-retest reliability.** Test-retest reliability of the clustering algorithm was plotted as a function of cluster number. Half of the resting runs were randomly selected from each subject and assigned to the test group; the other half were assigned to the re-test group. The reliability of the clustering algorithm was estimated as the mean similarity between the corresponding states derived from the test and re-test groups. This data splitting procedure was repeated 100 times. Mean reliability (black curve) across the 100 repetitions and standard error (the grey shadow area around the black curve) were plotted. Reliability decreased when the cluster number increased. Several local maxima on the curve were identified (marked with stars), suggesting that the solutions with 11, 14, 19, and 31, 35 clusters were relatively reliable. In this study, we focused on a 19-states estimation for further analyses.


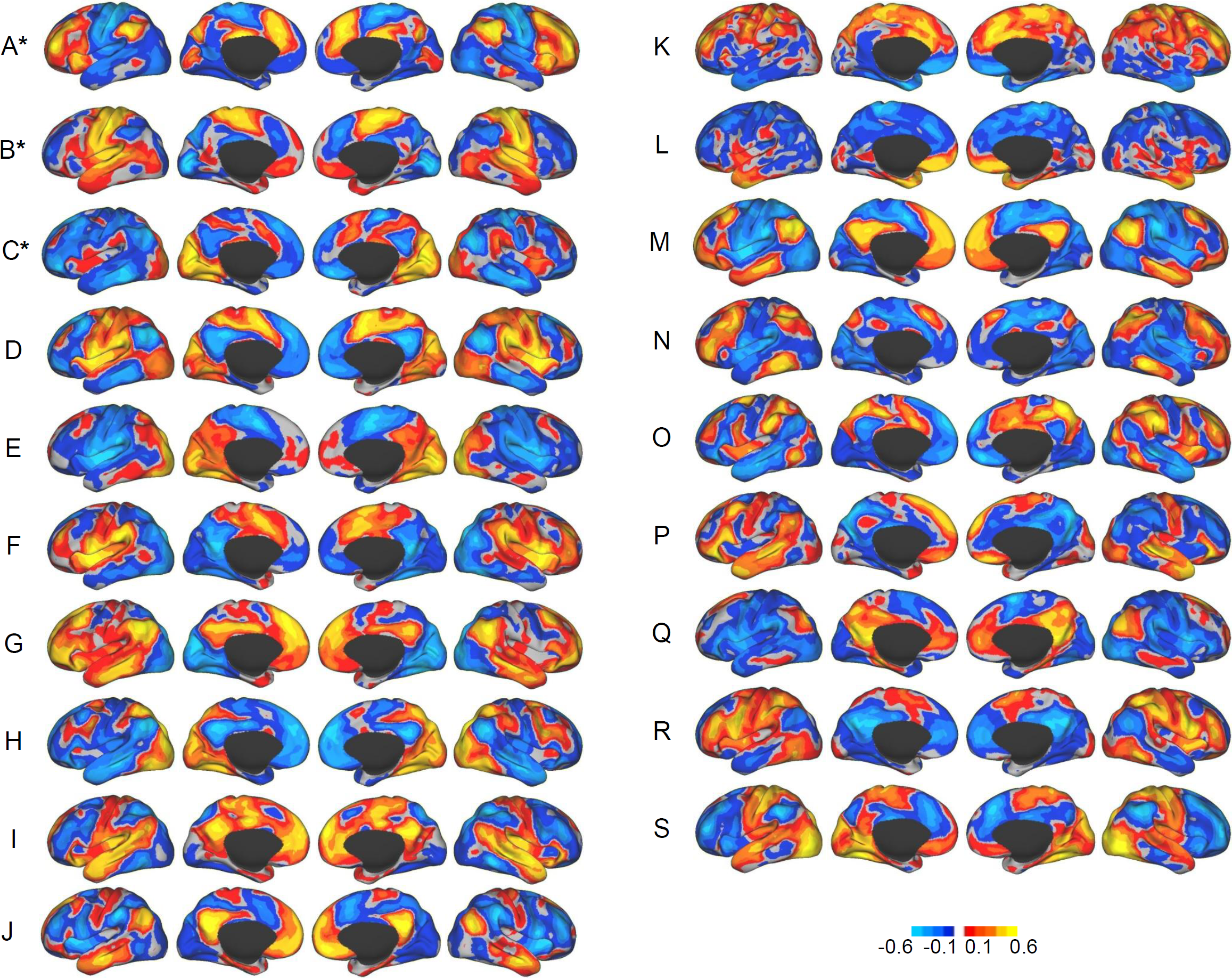


**Figure S2.** **Spatial maps of the 19 transient brain states.** The three states (A, B and C) which showed significantly lower rates of occurrence in the schizophrenia group compared to the healthy controls are labeled with asterisks.


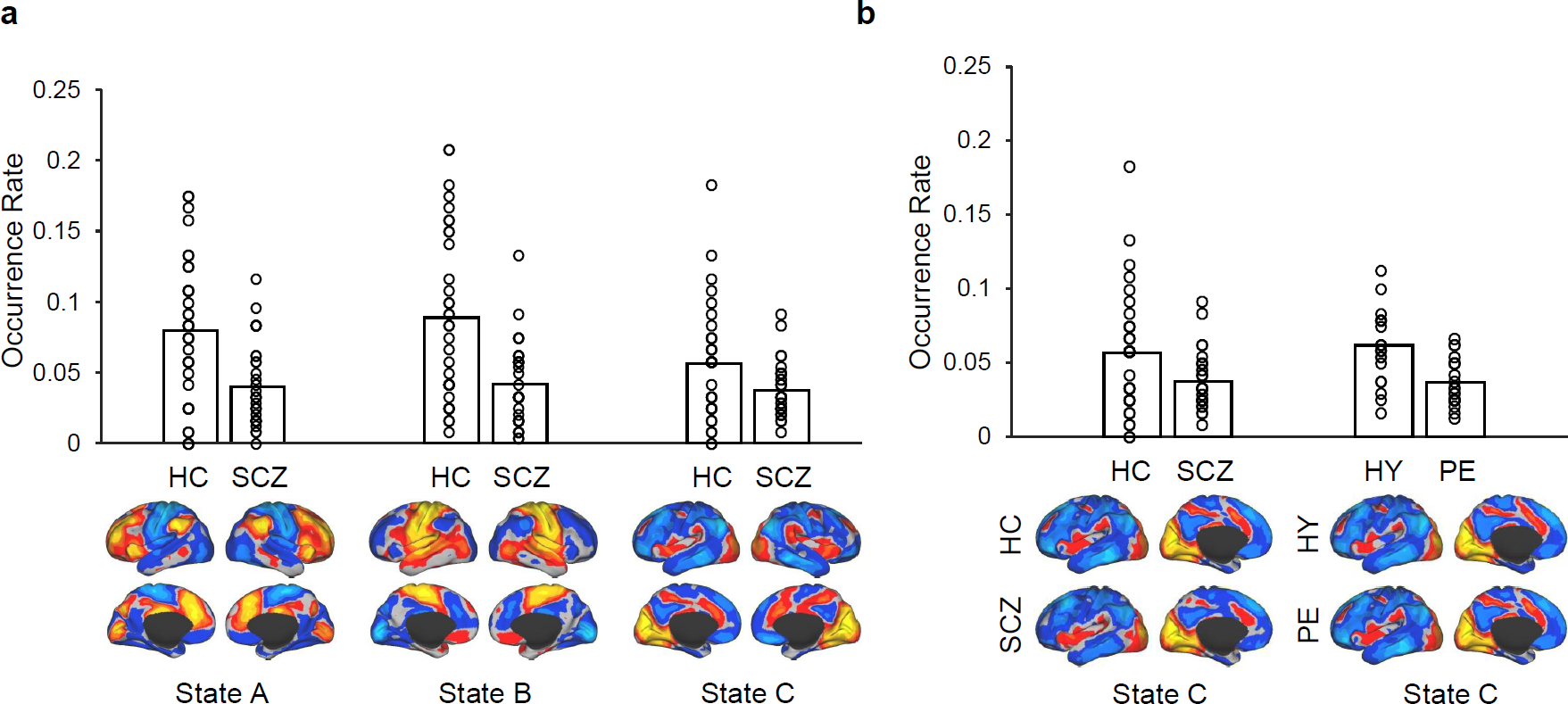


**Figure S3.** **Individual data of occurrence rates.** (a) The occurrence rates of three brain states were significantly reduced in SCZ patients, compared to healthy control subjects. These plots include the same data as displayed in Figure 2 of the main text but now display the data of individual subjects. Each subject is represented by a circle. (b) Occurrence rate of State C was reduced in PE compared to HY. This plot is the same as in main Figure 3 but shows individual data.


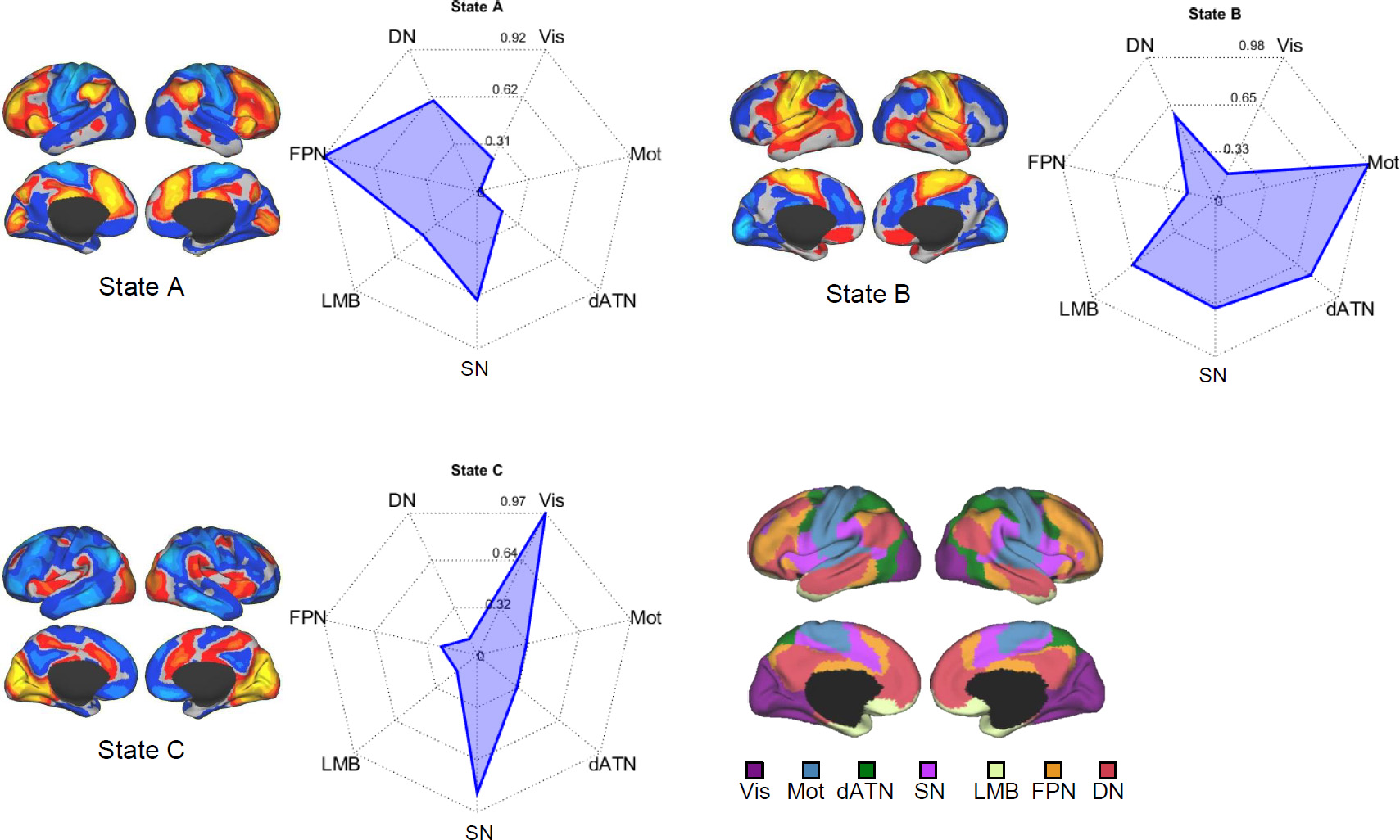


**Figure S4.** **Overlap between the maps of three brain states and 7 canonical networks.** For each brain state, the fingerprint plot demonstrates the percentage of area of each network that falls within the activated area of the state. State A is most overlapping with the frontoparietal network (FPN, Overlap = 0.92, i.e., 92% of the FPN was within the activated area in State A; overlap with VATN is 0.62; etc.), State B is most overlapping with the sensorimotor network (Mot, Overlap = 0.96), and State C is most overlapping with the visual network (Vis, Overlap = 0.97).


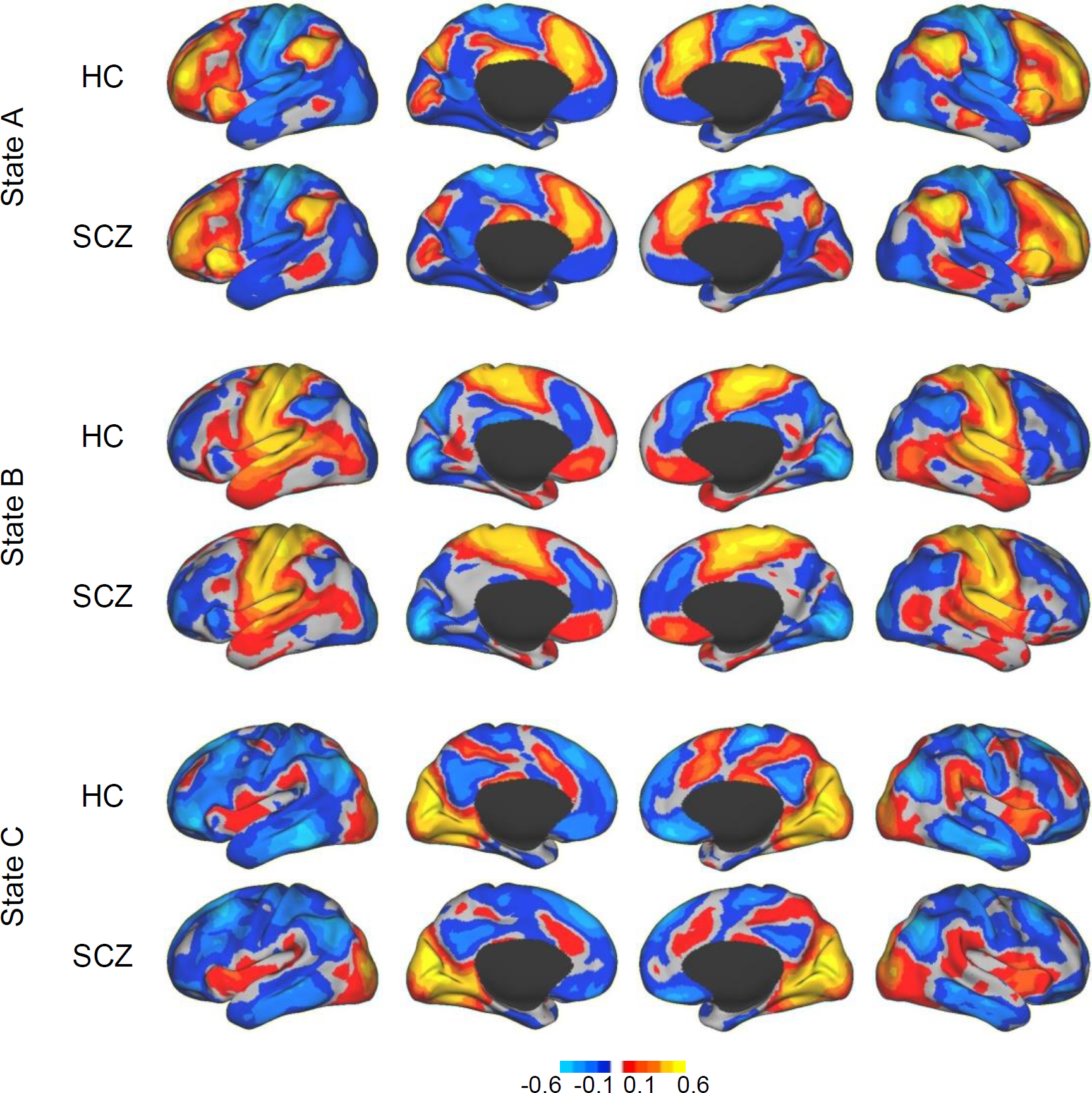


**Figure S5.** **Spatial distributions of brain states are intact in SCZ.** Spatial maps of the three brain states (A, B and C) that showed reduced rates of occurrence in SCZ showed high similarity between the SCZ and control groups (Spearman correlations: r = 0.94, 0.90, 0.92, respectively).


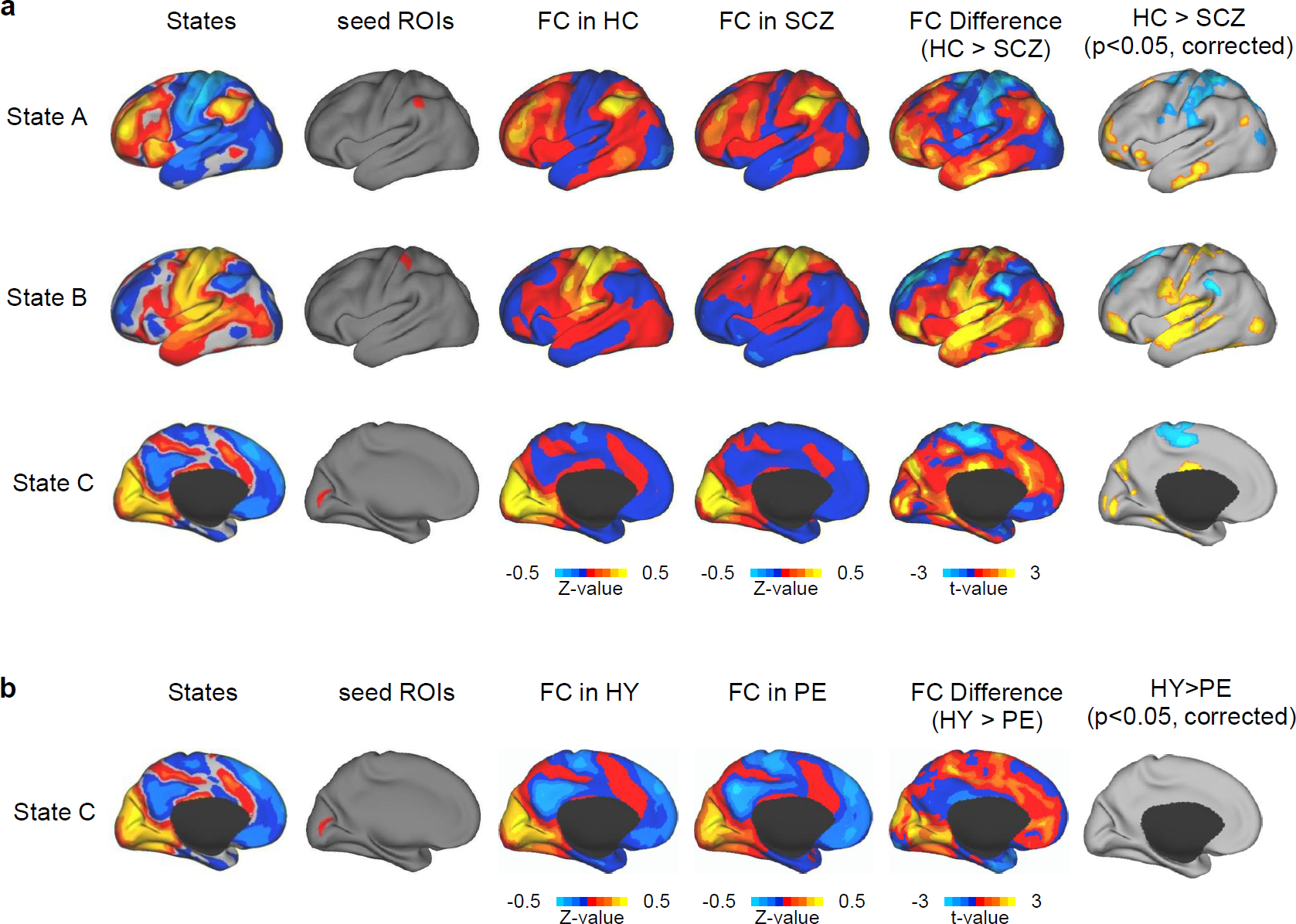


**Figure S6. Loss of dynamic states in SCZ and PE is associated with diminished “static” resting-state functional connectivity in the co-activated regions.** (a) Several brain regions that showed strong activity in brain states A, B and C (first column) were selected as the seeds (second column) for the generation of intrinsic functional connectivity (FC) maps (BOLD signal correlations). Connectivity maps based on these seeds (third and fourth columns) resembled the maps of dynamic brain states. Compared to HC, individuals with SCZ showed reduced connectivity between the seeds and specific regions (hot colors, fifth column). Regions with significantly reduced connectivity (FDR corrected for multiple comparisons at p < 0.05) are shown in the last column. (b) Compared to HY, individual with PEs show a trend towards a reduction in connectivity between the seed and co-activated regions in State C, but this difference was not significant after FDR correction.


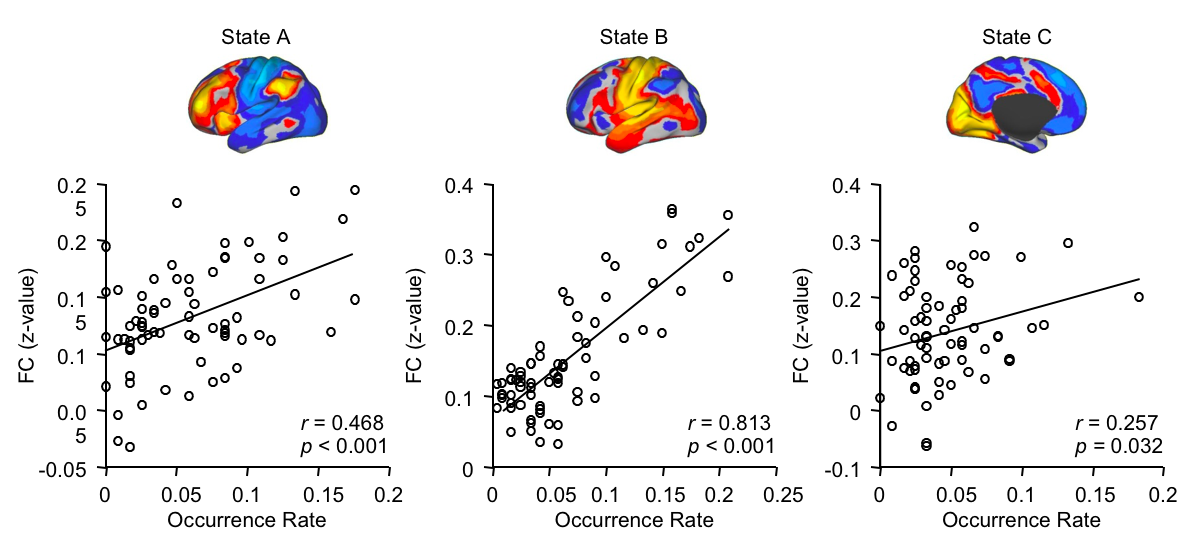


**Figure S7.** **Occurrence rates of States A, B and C are correlated with static connectivity between the seed ROI and the regions co-activated in these states.** Seed-based connectivity values were averaged within the mask of activated regions of the brain state, and then correlated with the rate of occurrence of that state. We found that rates of occurrence of State A, B and C were all significantly correlated with static connectivity within the brain state (State A: r = 0.468, p < 0.001; State B: r = 0.813, p < 0.001; State C: r = 0.257, p = 0.032).


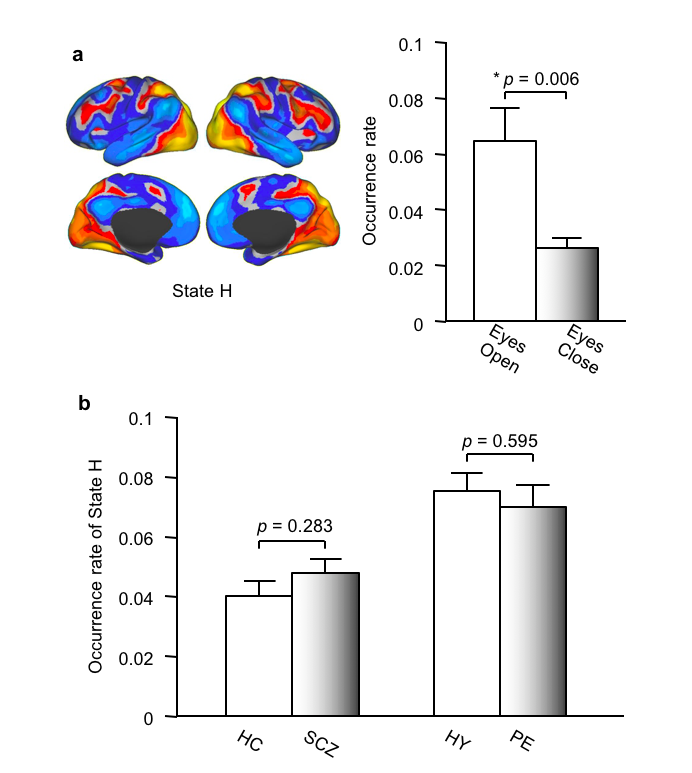


**Figure S8. The psychosis-related state is not affected by visual input.** (A) Nineteen states were derived from resting-state fMRI data collected in 20 healthy subjects that were scanned during rest with their eyes open (6 min) and with their eyes closed (6 min). Among the 19 states, only one state (state H) showed a significant difference (p=0.006) in occurrence rate between the “eyes open” and “eyes closed” conditions. This state (H) was comprised of the visual cortex, the dorsal attention network, and the frontoparietal control network. (B) Occurrence rate of this visual input-sensitive state (state H) was not significantly different between the SCZ and HC groups (p=0.283), and between the PE and HY groups (p=0.595), indicating that it was unlikely individuals with SCZ or PEs closed their eyes more frequently or were less attentive than controls.


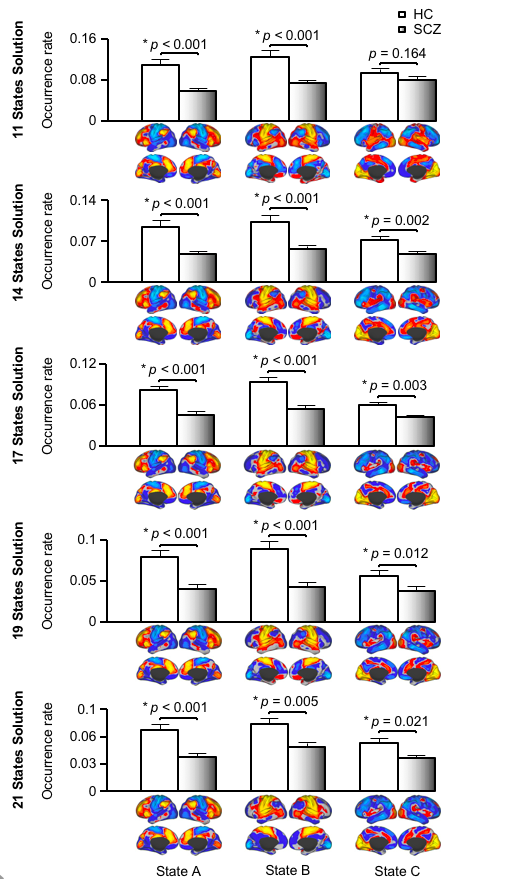


**Figure S9. Brain states derived from the 11-, 14-, 17-, 19- and 21-cluster solutions show consistent findings.** The three brain states (A, B and C) that showed significant differences in rates of occurrence between the SCZ and HC groups could be robustly reproduced when images were clustered into different numbers of brain states (i.e., 11, 14, 17, 19, 21), with one exception; in the 11-cluster solution, the difference between the two groups in occurrence rate of “State C” (which was altered in its distribution in the 11-cluster solution) was no longer significant. This was likely due to the fact that in the 11-cluster solution, State C represented a combination of the State C observed in the other solutions and additional sensorimotor-related states that were dissociable when a higher number of clusters was used.

**References**

1. Eryilmaz H, Tanner AS, Ho NF, Nitenson AZ, Silverstein NJ, Petruzzi LJ *et al.* Disrupted Working Memory Circuitry in Schizophrenia: Disentangling fMRI Markers of Core Pathology vs Other Aspects of Impaired Performance. *Neuropsychopharmacology* 2016; **41**(9)**:** 2411-2420.

2. First MB, Spitzer, RL, Gibbon, M, & Williams, JBW Structured Clinical Interview for DSM-IV Axis I Disorders, Research Version, Non-Patient Edition (SCID-I/NP). *New York Biometrics Research, New York State Psychiatric Institute* 2002.

3. Kay SR, Fiszbein A, Opler LA. The positive and negative syndrome scale (PANSS) for schizophrenia. *Schizophrenia bulletin* 1987; **13**(2)**:** 261-276.

4. Farabaugh A, Bitran S, Nyer M, Holt DJ, Pedrelli P, Shyu I *et al.* Depression and suicidal ideation in college students. *Psychopathology* 2012; **45**(4)**:** 228-234.

5. Holmes AJ, Hollinshead MO, O'Keefe TM, Petrov VI, Fariello GR, Wald LL *et al.* Brain Genomics Superstruct Project initial data release with structural, functional, and behavioral measures. *Sci Data* 2015; **2:** 150031.

6. Wang D, Buckner RL, Fox MD, Holt DJ, Holmes AJ, Stoecklein S *et al.* Parcellating cortical functional networks in individuals. *Nat Neurosci* 2015; **18**(12)**:** 1853-1860.

7. Durbin J, Watson GS. Testing for serial correlation in least squares regression. I. *Biometrika* 1950; **37**(3-4)**:** 409-428.

8. Wang C, Ong JL, Patanaik A, Zhou J, Chee MW. Spontaneous eyelid closures link vigilance fluctuation with fMRI dynamic connectivity states. *Proc Natl Acad Sci U S A* 2016; **113**(34)**:** 9653-9658.
